# Several first-line anti-hypertensives act on fibrosarcoma progression and PD1ab blockade therapy

**DOI:** 10.1186/s13018-024-04627-w

**Published:** 2024-02-19

**Authors:** Jianwen Sun, Chaoxiong Zhang, Xinhao Su, Haoyun Zhou, Siyun Zhou, Minjie Jiang, Binbo Fang

**Affiliations:** 1 Department of Orthopaedics, The First Affiliated Hospital of Jishou University, The People’s Hospital of Xiangxi Autonomous Prefecture, Jishou, China; 2 Department of Medicine, Taizhou University, Zhejiang, China; 3 Department of Jishou University, Jishou, China

**Keywords:** Anti-hypertensive, PD1ab, Captopril, Verapamil, Hydrochlorothiazide, SLC12A3, Fibrosarcoma

## Abstract

**Purpose:**

Patients are typically diagnosed with both hypertension and fibrosarcoma. Medical oncologists must prescribe suitable anti-hypertensive medications while considering anti-tumor drugs. Recently, immunotherapy has become prominent in cancer treatment. Nonetheless, it is unknown what role anti-hypertensive medications will play in immunotherapy.

**Methods:**

We examined the effects of six first-line anti-hypertensive medications on programmed cell death protein 1 antibody (PD1ab) in tumor treatment using a mouse model of subcutaneous fibrosarcoma. The drugs examined were verapamil, losartan, furosemide, spironolactone, captopril, and hydrochlorothiazide (HCTZ). The infiltration of CD8^+^ T cells was examined by immunohistochemistry. Additionally, several in vitro and in vivo assays were used to study the effects of HCTZ on human fibrosarcoma cancer cells to explore its mechanism.

**Results:**

Verapamil suppressed tumor growth and showed an improved effect on the tumor inhibition of PD1ab. Captopril did not affect tumor growth but brought an unexpected benefit to PD1ab treatment. In contrast, spironolactone and furosemide showed no effect on tumor growth but had an offset effect on the PD1ab therapy. Consequently, the survival time of mice was also significantly reduced. Notably, losartan and HCTZ, especially HCTZ, promoted tumor growth and weakened the effect of PD1ab treatment. Consistent results were observed in vivo and in vitro using the human fibrosarcoma cell line HT1080. We determined that the Solute Carrier Family 12 Member 3 (SLC12A3), a known target of HCTZ, may be the principal factor underlying its effect-enhancing properties through mechanism studies employing The Cancer Genome Atlas (TCGA) data and in vivo and in vitro assays.

**Conclusion:**

Verapamil and captopril potentiated the anti-tumor effect of PD1ab, whereas spironolactone and furosemide weakened the effect of PD1ab on tumor inhibition. Alarmingly, losartan and HCTZ promoted tumor growth and impaired the effect of PD1ab. Furthermore, we preliminarily found that HCTZ may promote tumor progression through SLC12A3. Based on this study, futher mechanism researches and clinical trials should be conducted in the future.

## Introduction

Fibrosarcoma is a type of malignant neoplasm originating from mesenchymal cells; it can occur in any part of the human body containing fibrous tissue [1]. Hypertension is a global problem. The likelihood of co-diagnosing both diseases in a single patient is high. Hypertension is closely related to the risk of most common malignant tumors, such as colon, oral, lung, laryngeal, and esophageal cancers [2–5]. The mortality rate of patients with hypertension is significantly higher than that of patients with normal blood pressure in all cancer types, especially in patients with renal cell carcinoma, oral cancer, and small lung cancer [3, 6, 7]. Thus, it is important to regulate the blood pressure of patients with cancer.

Different anti-hypertensive medications have varying effects on cancer risk. Verapamil has been reported to restrain tumor progression and decrease cancer risk or mortality [8, 9], but it increases the risk of numerous cancer types [10–12]. Controversial reports continue to surround the use of captopril in tumor progression, suggesting that it may increase [13, 14] or decrease [15] the risk of cancer or cancer progression. Spironolactone has also been reported to decrease [16, 17] or show no significant impact [18] on cancer risk. Losartan has different outcomes for the risk of various cancers [19, 20]. Hydrochlorothiazide (HCTZ), one of the most frequently used diuretic and anti-hypertensive drugs, is associated with an increased risk of non-melanoma skin malignancy due to its photosensitizing properties [21]. HCTZ exerts its effect by acting on the proximal region of the distal convoluted tubule, suppressing reabsorption by regulating the sodium-chloride symporter, also known as Solute Carrier Family 12 Member 3 (SLC12A3) [22–24]. SLC12A3 inhibition reduces the magnitude of the concentration gradient between the distal convoluted tubule and epithelial cells, thereby impeding water reabsorption [22].

Immunotherapy for cancer has recently taken a massive step forward and has become a sharp sword for cancer therapy. Currently, checkpoint blockade is the immunotherapy class with the most advanced research. Blockade of the programed death-1/programed death-ligand 1 (PD-1/PD-L1) axis is one of the most common checkpoint inhibition strategies. In the tumor microenvironment, to escape recognition and elimination by T cells, tumor cells increase PD-L1 expression, bind to PD-1 on T cells, and suppress their function [25–27]. The clinical application of PD-1/PD-L1 checkpoint blockade has expanded rapidly in recent years. Five PD1ab or PD-L1ab products have been approved for clinical use in cancer therapy, offering a better prognosis and reduced toxicity compared with conventional chemotherapies [28]. However, whether anti-hypertensive drugs exert synergistic, neutral, or antagonistic effects on PD-1/PD-L1 checkpoint blockade remains unclear. This study aimed to assist medical oncologists in comprehending and selecting evidence-based anti-hypertensive medications, particularly when using PD-1/PD-L1 inhibitors in oncotherapy.

In this study, we investigated the effects of six first-line anti-hypertensive drugs, including verapamil, captopril, spironolactone, HCTZ, losartan, and furosemide, on PD1ab using a mouse fibrosarcoma cancer model. Our results revealed that verapamil impaired tumor proliferation ability and enhanced the therapeutic efficacy of PD1ab. Captopril was also a favorable factor for PD1ab. Furthermore, spironolactone and furosemide weaken the efficacy of PD1ab on tumor growth. Notably, HCTZ significantly promoted tumor growth by regulating SLC12A3 and weakening the therapeutic effect of PD1ab.

## Materials and methods

### Cell lines

The murine fibrosarcoma cell line MCA-205 and human fibrosarcoma cell line HT1080 were purchased from Shanghai Hongshun Biotechnology and originally acquired from the American Type Culture Collection (ATCC), which also provided their STR identification report. The MCA-205 and HT1080 cell lines were cultured in DMEM and RPMI 1640, respectively, with 10% fetal bovine serum (FBS) in an incubator at 37 °C with 5% CO_2_. Both cell lines underwent a mycoplasma test using MycAway™ Mycoplasma Real-time qPCR Detection Kit.

### Ethical approval and informed consent

All animal experiments were conducted in accordance with the guidelines for animal handling provided by the Care and Use of Laboratory Animals of the National Institutes of Health (NIH). The tumor burden in mice did not exceed the recommendations of the Institutional Animal Care and Use Committee of the University of Pennsylvania. The procedure was approved by the Animal Care and Use Committee of Tai Zhou University (Approval number: TZXY-2023–20231065).

To construct subcutaneous models, C57BL/6 J, BALB/c, and nude mice (5–6 weeks, 19–20 g) were used. The MCA-205 and HT1080 cell lines were cultured at 90% confluence to prepare a cell suspension with 0.1–0.5 × 10^6^ cells/100 μL and 0.5–1 × 10^6^ cells/100 μL. Subcutaneous injections were administered to the left dorsal flanks of each mouse. The subcutaneous tumor grew palpably after approximately 6–8 days. A slide caliper was employed to measure tumor length including base diameter (A) and perpendicular value (B). The formula used to calculate the tumor mass was 0.5 *A *B^2^. The mice were sacrificed when the tumor reached approximately 1000 mm^3^ or when evident ulceration occurred approximately 14–16 days after cell injection. Survival of mice was monitored biweekly and euthanized when the tumor area reached > 1500 mm^3^.The subcutaneous tumors were then surgically removed and preserved in formalin (10%) for at least 48 h.

Regarding the therapy regimen, based on previous literature, the mice were administered 250 µg PD1ab (BE0146, BioXCell, i.p.) or IgG isotype control (BE0089, BioXCell, i.p.), on days 6–7 after cell injection, every other day, with a total dose limited to 1 mg per mouse. Anti-hypertensive drugs, including hydrochlorothiazide (100 mg/kg, MedChemExpress), captopril (50 mg/kg, MedChemExpress), losartan (40 mg/kg, MedChemExpress), verapamil (25 mg/kg, MedChemExpress), spironola-ctone (5 mg/kg, MedChemExpress), furosemide (200 mg/kg, MedChemExpress) and dimethyl sulfoxide (DMSO), were intraperitoneally administered every other day.

### Immunohistochemistry

The tumor sections were heated, deparaffinized, rehydrated, and immersed in sodium citrate buffer (pH = 6.0). When endogenous peroxidase activity interfered, the slides were treated with xylene, and 3% hydrogen peroxide was added. After three washes with PBS, the sections were incubated with the primary antibody (Cat#ab209775, ABCAM, rabbit anti-CD8, 1:1000 dilution, 4 °C, 24 h) according to the manufacturer’s instructions. A secondary antibody (Cat#7074, Cell Signaling, anti-rabbit IgG, 1:2000 dilution, 1 h, 37 °C) was added after washing with PBS. After successful staining with 3,3-diaminobenzidine (DAB), the sections were washed three times with PBS, counterstained with Mayer’s hematoxylin, dehydrated, and mounted. Finally, the slides were photographed, and the positive cells were counted calculated in six randomly selected fields (×200) under a microscope.

### Western blot analysis

Proteins isolated from the targeted cells were lysed, separated by SDS–polyacrylamide gel electrophoresis (PAGE), and electrotransferred into a standard polyvinylidene difluoride (PVDF) membrane. Primary antibodies that target SLC12A3 (1:1000 dilution, ab95302) were used to block the membranes with 10% skimmed milk and incubated at 4 °C for a minimum of 24 h. After washing the membranes five times for 10 min each with TBST, they were incubated with the secondary antibody for 1–2 h at 37 °C. After three washes with TBST for 10 min each, bands were detected using an enhanced chemiluminescence detection system (Amersham Biosciences Europe, Freiberg, Germany).

### Cell proliferation assay

1 × 10^3^ cells were seeded on 96-well plates and cultured at 37 °C under a humidified atmosphere with 5% CO_2_ for 24 h. 2-(2-Methoxy-4-nitrophenyl)-3-(4-nitrophenyl)-5-(2,4-disulfothenyl)-2H-tetrazolium salt (CCK-8, Dojindo, Rockville, USA) solution was added to each well and incubated for 1 h, the absorbance was measured at 450 nm with a Microplate Autoreader (Bio-Rad, Hercules, CA, USA). The experiment was performed with three replicates.

For colony formation assay, 600 cells were seeded on six-well plates and cultured in an incubator for 7–10 days. Colonies were stained with Giemsa dye for 30 min after fixation with 4% paraformaldehyde. Each well was photographed. Then, the number of colonies was counted (defined as > 50 cells/colony) using Image J software. The experiment was repeated three times.

### Cell wound healing assay

In total, 1–2 × 10^6^ cells were seeded into six-well plates and incubated for 1–2 days until the surface of the well was completely covered with cells. Scratch wounds were produced using a suitable pipette. Images were captured in three randomly selected microscopic fields (×200) between the advancing margins to estimate the level of migration in each group of cells at 0, 12, 24, and 36 h, singly. Cell motility was determined and assessed in serum-free media by counting the number of cells.

### Transwell migration assay

The number of 1–2 × 10^5^ cells per well was determined and transferred to the upper compartment of transwells filled with serum-free media, whereas the lower compartment was filled with media containing 10% FBS. The cells were cultured in an incubator for 24 h. The wells with successful cell translocation were then fixed with 4% paraformaldehyde. Giemsa dye was used to stain the cells for 30 min, and the number of cells was calculated in five randomly selected fields (×200) under a microscope.

### Enzyme-linked immunosorbent assay (ELISA)

Vascular endothelial growth factor (VEGF) levels were quantified using a VEGF enzyme-linked immunosorbent assay kit. The fibrosarcoma cell line MCA-205 was cultured in a medium containing 10 μM concentration of various anti-hypertensive drugs, including verapamil, losartan, furosemide, spironolactone, captopril and HCTZ for 24 h. The medium was collected and analyzed according to the manufacturer’s instructions, and quality control was ensured. The experiments were repeated three times.

### Lentiviral transduction and transfection

The human shRNA sequences that inhibit SLC12A3 expression are listed as follows: NC:CCTAAGGTTAAGTCGCCCTCGCTCGAGCGAGGGCGACTTAACCTTAGG. shRNA#1:CAGGAGAGAAAGGCGATCATT.shRNA#2:GAGACCTTCATTCCAA(Cyagen, Suchow China). HEK293 cells were transfected with the above-mentioned vectors to produce targeted recombinant lentiviruses as a package tool. The media was used to transfect HT1080 cells. After continuous use of 0.5 mg/mL puromycin for at least 7–10 days, additional validation was performed using western blotting analyses.

The steadily transduced cells were selected.

### Bioinformatic analysis

Clinical data sarcoma patients were downloaded from the TCGA database by utilizing the UCSC Xenia (https://xenabrowser.net/). The values of SLC12A3 expression were estimated by Log2 transcripts per kilo base million value. The Cumulative Survival was generated from TCGA. SLC12A3 expression between normal tissues and sarcoma tissues were statistically compared by* t*-test*.*

### Statistical analyses

Results are shown as the mean ± SD. The SPSS 19.0 software package was used to assess the difference between two groups or two groups at different time points through nonparametric tests, specifically two-way ANOVA or two-tailed *t*-test. *P* < 0.05 was considered statistically significant.

## Results

The same isotype and PD1ab controls were used for all subcutaneous tumor growth assays, and tumors were harvested simultaneously.

### Verapamil inhibited fibrosarcoma growth and enhanced immune responses to PD1ab

**Fig. 1:**
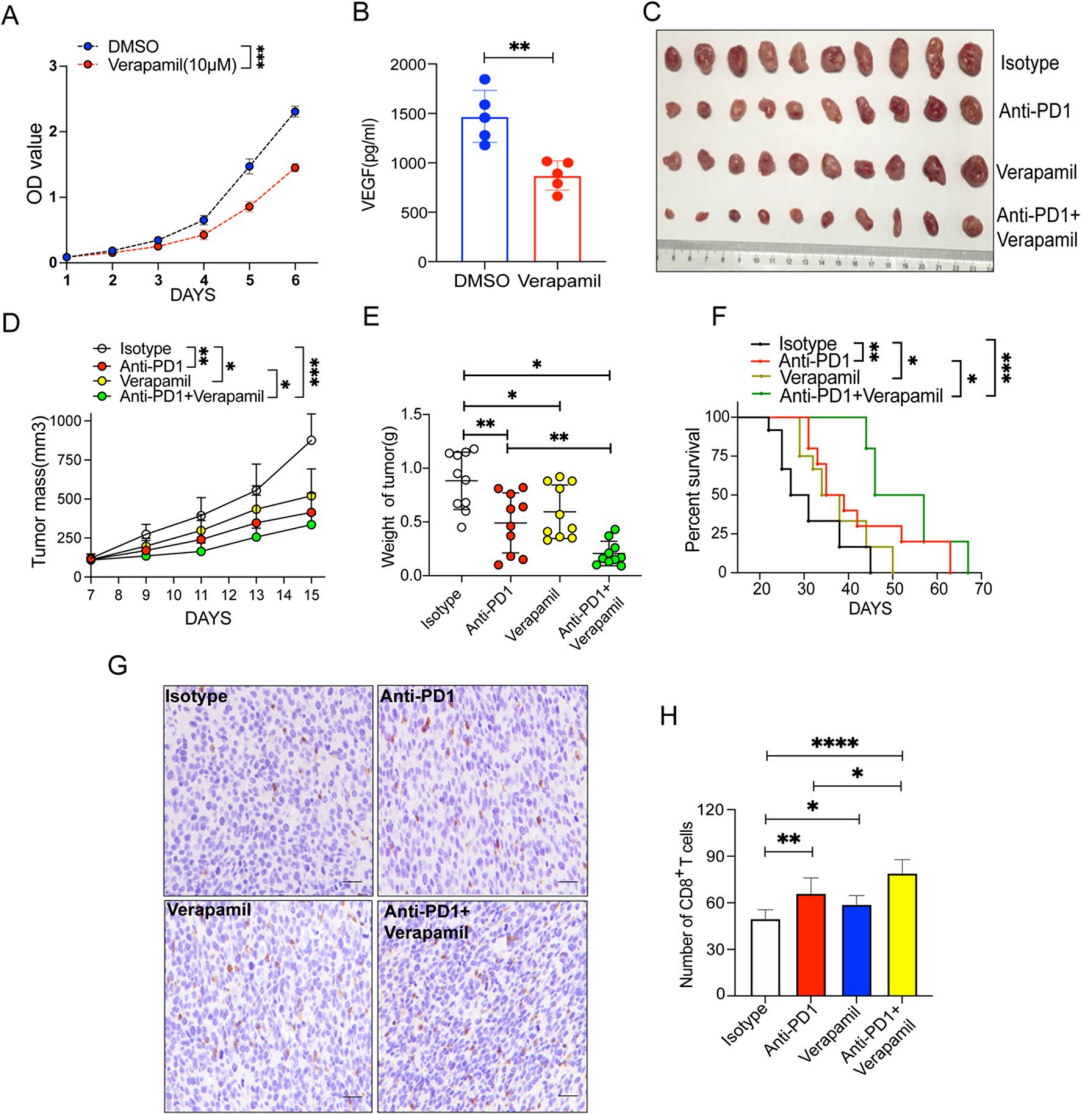
Verapamil showed enhanced effects on fibrosarcoma and tumor immune responses to PD1ab. **A** CCK8 experiment was used to detect the proliferation ability of MCA-205 after verapamil treatment. **B** Elisa kit was used to detect the VEGF production after verapamil treatment. **C** A double therapy (verapamil and PD1ab) or monotherapies were used for curing MCA-205 neoplasms on mice. **D**–**F** Tumor mass (**D**) and weight (**E**) from MCA-205-neoplasm mice (n = 10) were demonstrated. **F** Survival of mice (n = 10) was monitored biweekly after treatment. **G**–**H** The level of CD8^+^ T cells infiltration after treatment. Scale bars, 20 μm

Verapamil is widely used in clinical practice as a non-dihydropyridine calcium channel blocker. In vitro, verapamil significantly inhibited tumor growth (Fig. 1A). Additionally, verapamil significantly decreased VEGF production in the MCA-205 cell line (Fig. 1B). In the murine subcutaneous fibrosarcoma cancer model, verapamil monotherapy significantly inhibited growth and enhanced immune responses to PD1ab (Fig. 1C–E). In MCA-205 tumor-bearing mice, the group that received verapamil monotherapy and the combination with PD1ab had a longer survival time (Fig. 1F). The results suggested that the in vitro action of verapamil on tumor cells could be translated into an in vivo effect. Immunohistochemistry examination revealed that verapamil had a greater impact on the number of tumor-infiltrated CD8^+^ T cells than the control, and the combination with PD1ab induced more infiltration of CD8^+^ T cells (Fig. 1G–H).

### Captopril had no effect on fibrosarcoma growth but enhanced tumor immune responses to PD1ab

**Fig. 2:**
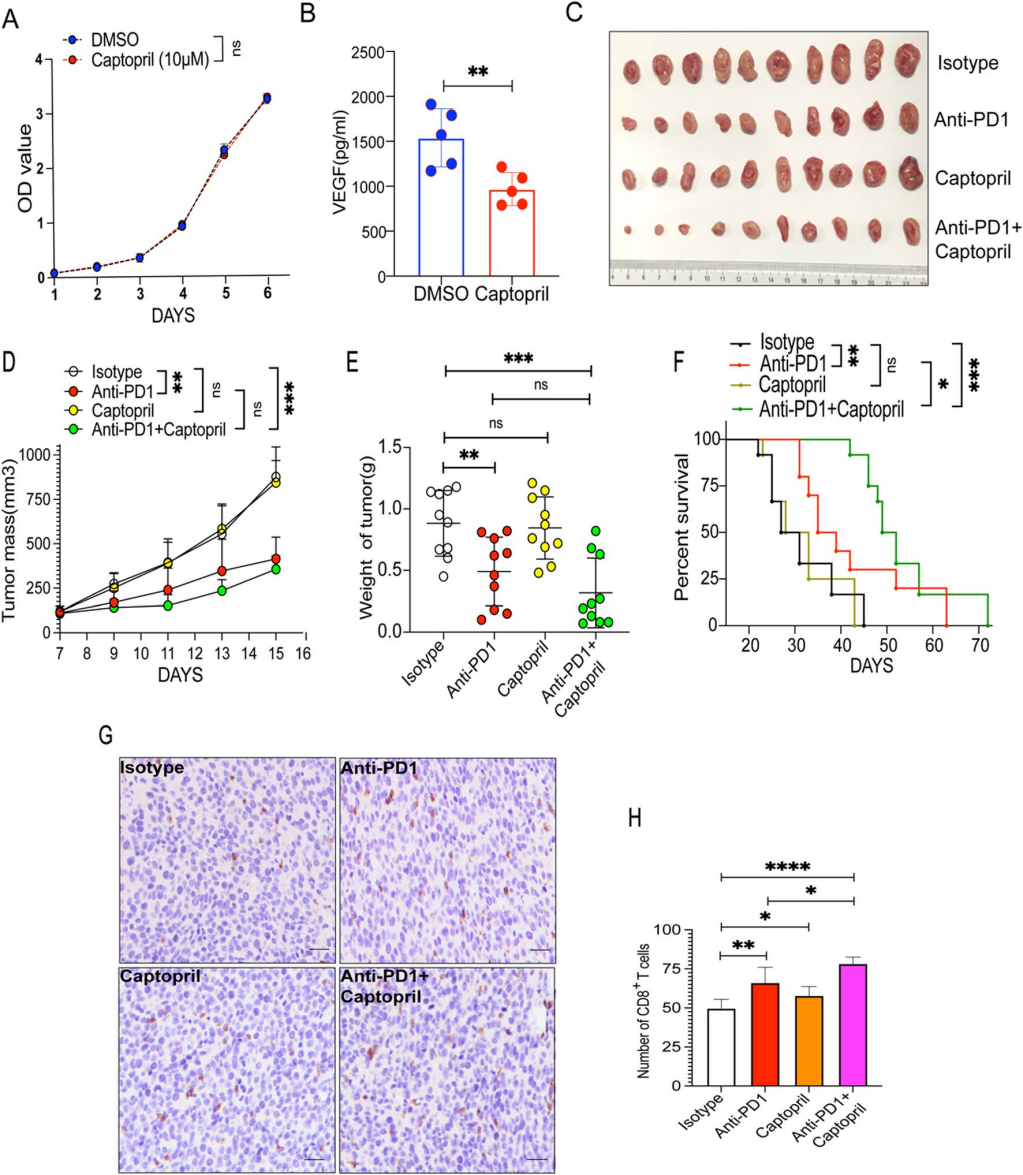
Captopril showed no effect on fibrosarcoma growth but enhanced tumor immune responses to PD1ab. **A** CCK8 experiment was used to detect the proliferation ability of MCA-205 after captopril treatment. **B** Elisa kit was used to detect the VEGF production after captopril treatment. **C** A double therapy (captopril and PD1ab) or monotherapies (captopril or PD1ab) were used for curing MCA-205-neoplasms on mice. **D**–**F** Tumor mass (**D**) and weight (**E**) from MCA-205-neoplasms mice (n = 10) were demonstrated. **F** Survival of mice (n = 10) was monitored biweekly after treatment. **G**–**H** The level of CD8^+^ T cells infiltration after treatment. Scale bars, 20 μm

Captopril is a widely used angiotensin-converting enzyme inhibitor (ACEI). It reduces plasma angiotensin II levels and decreases aldosterone secretion and vasodilation [29]. In vitro, captopril had no effect on fibrosarcoma growth (Fig. 2A). However, it significantly reduced VEGF production from MCA-205 cell line (Fig. 2B). In a murine subcutaneous fibrosarcoma model (Fig. 2C), PD1ab significantly suppressed the growth of MCA-205 fibrosarcoma in wild-type mice, while captopril did not significantly affect MCA-205 tumor growth. The combination therapy of captopril and PD1ab exhibited enhanced therapeutic effectiveness on MCA-205 fibrosarcoma compared with PD1ab single therapy (Fig. 2D–E). Administration of captopril in conjunction with PD1ab resulted in a significant increase in survival (Fig. 2F). Immunohistology examination revealed that, compared with control and PD1ab treatment, captopril and the combination of both agents increased the infiltration of CD8^+^ T cells, respectively (Fig. 2G–H).

### *Losartan showed different effects on fibrosarcoma in the *in vitro* and *in vivo* experiments*

**Fig. 3:**
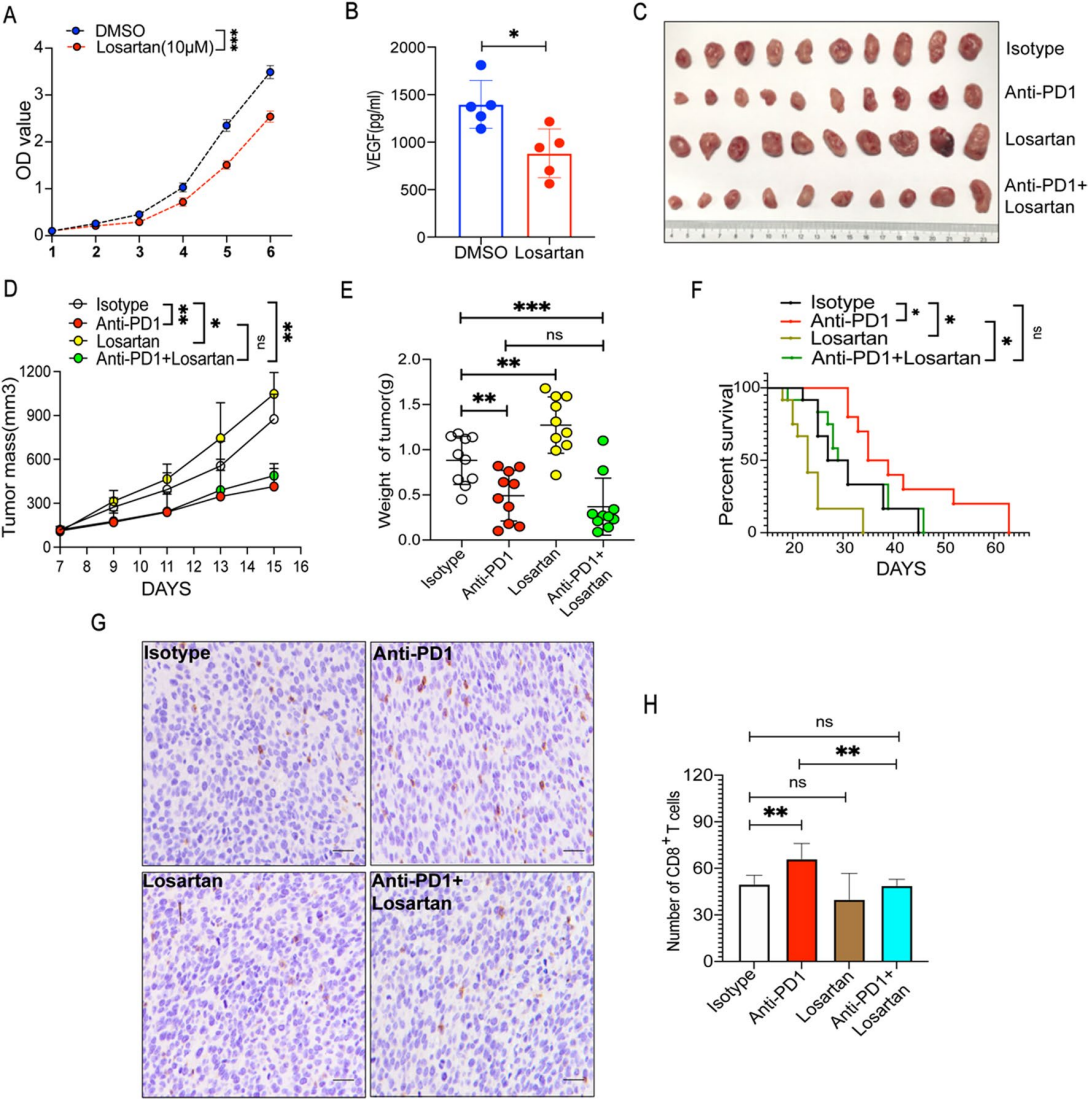
Losartan showed different effects on fibrosarcoma in vitro and in vivo. **A** CCK8 experiment was used to detect the proliferation ability of MCA-205 after losartan treatment. **B** Elisa kit was used to detect the VEGF production after losartan treatment. **C** A double therapy (losartan and PD1ab) or monotherapies (losartan or PD1ab) were used for curing MCA-205-neoplasms mice. **D**–**F** Tumor mass (**D**) and weight (**E**) from MCA-205-neoplasms mice (n = 10) were demonstrated. **F** Survival of mice (n = 10) was monitored biweekly after treatment. **G**–**H** The level of CD8^+^ T cells infiltration after therapy after treatment. Scale bars, 20 μm

Losartan, a selective and competitive angiotensin II receptor blocker, exerts its effect by inhibiting the release of adrenal catecholamine angiotensin II-induced vasopressin, resulting in a rapid and durable effect on lowering blood pressure. Additionally, losartan can inhibit the action of aldosterone and angiotensin II-induced vasoconstriction, thus lowering blood pressure [30]. In vitro, losartan significantly suppressed tumor growth (Fig. 3A), and the amounts of VEGF in the supernatant decreased after treatment (Fig. 3B). In MCA-205 fibrosarcoma-bearing mice (Fig. 3C), unexpectedly, losartan monotherapy promoted the proliferation of MCA-205 fibrosarcoma cells in vivo. Compared with PD1ab single therapy, the combination therapy of losartan and PD1ab did not inhibit MCA-205 progression (Fig. 3D–E). However, the addition of losartan deprived the survival benefit of PD1ab (Fig. 3F). The tumor microenvironment may be responsible for the phenomenon. The immunohistochemistry analysis found that PD1ab significantly augmented the number of tumoral infiltrated CD8^+^ T cells compared with the control, while losartan significantly decreased. Compared with PD1ab single therapy, the combination therapy of losartan and PD1ab decreased CD8^+^ T cell infiltration (Fig. 3G–H).

### Furosemide did not affect fibrosarcoma growth but weakened tumor immune responses to PD1ab

**Fig. 4:**
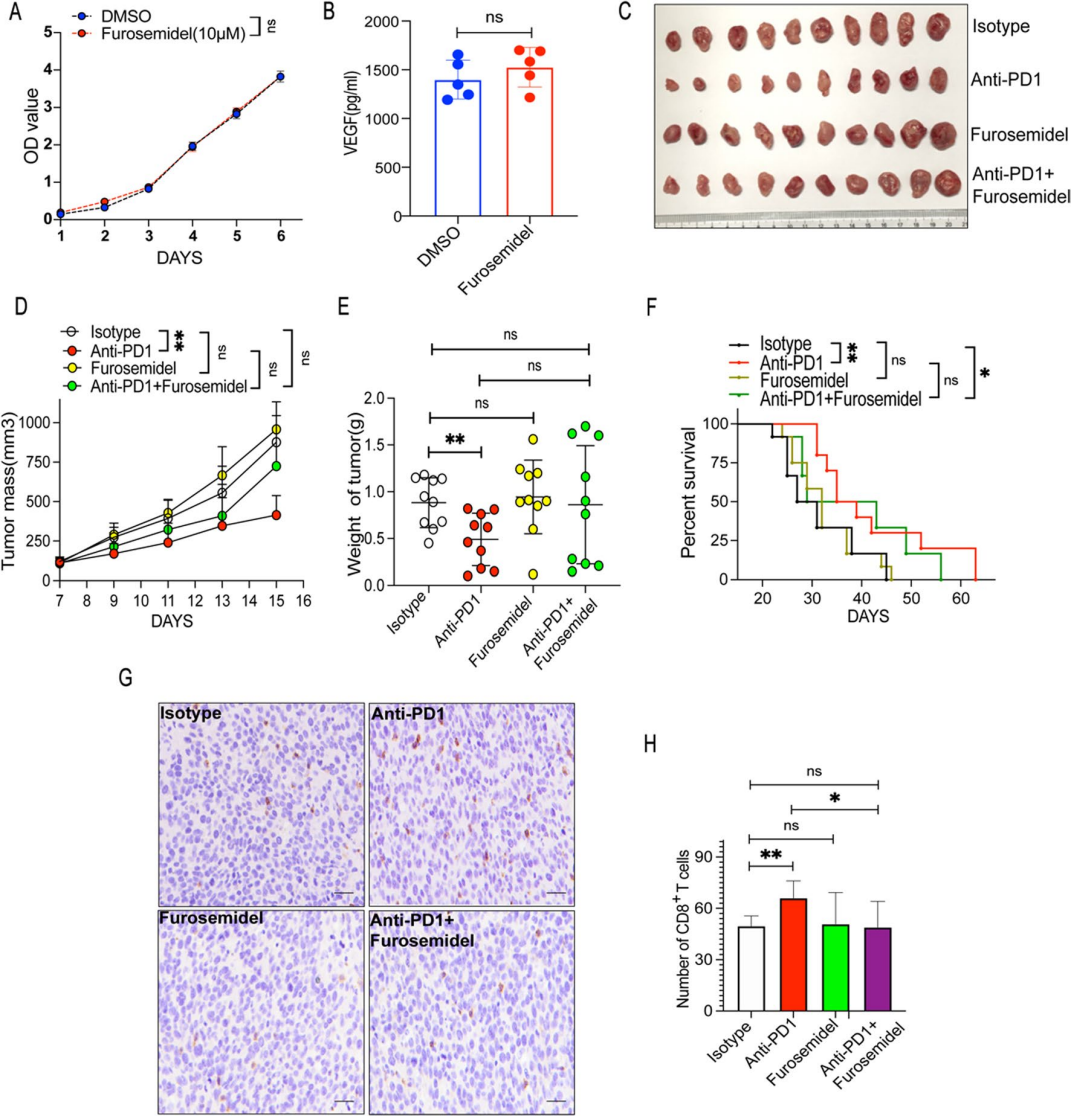
Furosemide showed no effect on fibrosarcoma growth but weakened tumor immune responses to PD1ab. **A** CCK8 experiment was used to detect the proliferation ability of MCA-205 after furosemide treatment. **B** Elisa kit was used to detect the VEGF production after furosemide treatment. **C** A double therapy (furosemide and PD1ab) or monotherapies (furosemide or PD1ab) were used for curing MCA-205-neoplasms mice. **D**–**F** Tumor mass (**D**) and weight (**E**) from MCA-205-neoplasms mice (n = 10) were demonstrated. (**F**) Survival of mice (n = 10) was monitored biweekly after treatment. **G**–**H** The level of CD8^+^ T cells infiltration after treatment. Scale bars, 20 μm

Furosemide, a loop diuretic, has been utilized in clinical practice for decades. Furosemide inhibits chloride, and tubular sodium reabsorption in the proximal tubule, the thick ascending loop of Henle and distal tubules [31]. In vitro, furosemide had no effect on MCA-205 growth (Fig. 4A) and did not significantly alter the level of VEGF produced by MCA-205 (Fig. 4B). In MCA-205 fibrosarcoma-bearing mice (Fig. 4C), furosemide did not inhibit MCA-205 fibrosarcoma growth. However, the combination of furosemide and PD1ab displayed a weaker therapeutic impact on tumor growth when compared with PD1ab single therapy (Fig. 4D–E). Consequently, furosemide monotherapy did not improve the survival of MCA-205 fibrosarcoma-bearing mice; mice that recieved the combination treatment of furosemide and PD1ab had a shorter survival time than those receiving PD1ab alone (Fig. 4F). According to immunohistological examination, furosemide showed no impact on the level of infiltrating CD8^+^ T cells compared with control. Compared with PD1ab treatment alone, the combination of both agents showed a decreasing impact on the level of infiltrating CD8^+^ T cells (Fig. 4G–H).

### Spironolactone did not affect fibrosarcoma growth but weakened tumor immune responses to PD1ab

**Fig. 5:**
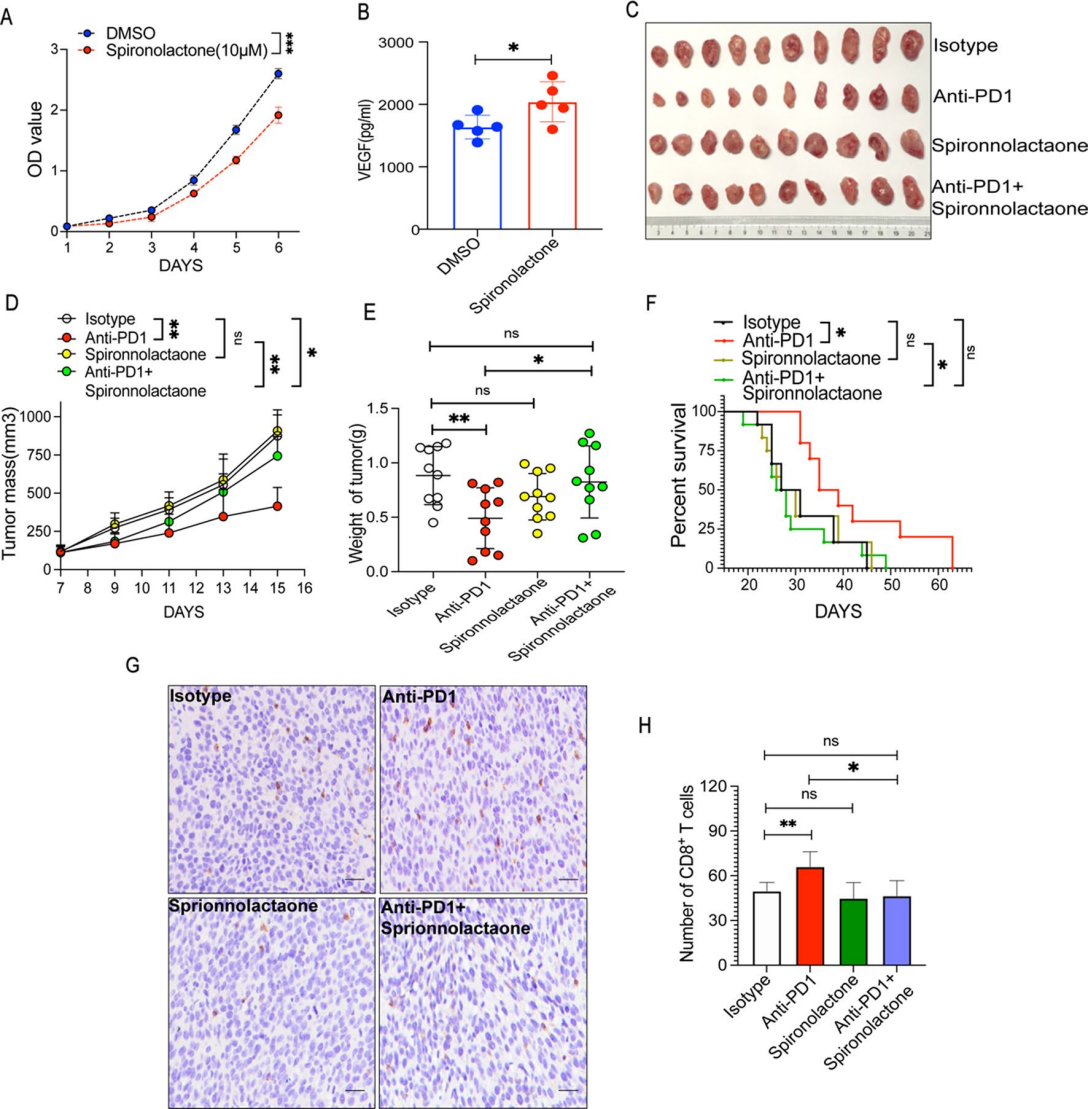
Spironolactone did not affect fibrosarcoma growth but weakened tumor immune responses to PD1ab. **A** CCK8 experiment was used to detect the proliferation ability of MCA-205 after of spironolactone treatment. **B** Elisa kit was used to detect the VEGF production after spironolactone treatment. **C** A double therapy (spironolactone and PD1ab) or monotherapies (spironolactone or PD1ab) were used for curing MCA-205-neoplasms mice. **D**–**F** Tumor mass (**D**) and weight (**E**) from MCA-205-neoplasms mice (n = 10) were demonstrated. **F** Survival of mice (n = 10) was monitored biweekly after treatment. **G**–**H** The number of CD8^+^ T cells infiltration after treatment. Scale bars, 20 μm

As a class of mineralocorticoid receptor antagonists, spironolactone is an effective first-line drug for hypertension. Spironolactone competitively blocks aldosterone receptor-mediated activation [32]. In vitro, spironolactone significantly inhibited tumor cell growth (Fig. 5A); however, it increased the amounts of VEGF in the supernatant after treatment (Fig. 5B). In MCA-205 fibrosarcoma-bearing mice, spironolactone did not inhibit tumor growth(Fig. 5C). However, the combination of spironolactone with PD1ab showed a weak therapeutic effect on tumor growth compared with PD1ab single therapy (Fig. 5D–E). As a result, spironolactone did not improve the survival of MCA-205 fibrosarcoma-bearing mice; rather, it diminished the survival time benefit conferred by PD1ab (Fig. 5F). On the basis of the immunohistological examination, spironolactone did not show any effect on the level of infiltrating CD8^+^ T cells. However, compared with PD1ab treatment alone, the combination of both agents reduced infiltrating CD8^+^ T cells (Fig. 5G–H).

### HCTZ promoted fibrosarcoma growth and reduced tumor immune responses to PD1ab

**Fig. 6:**
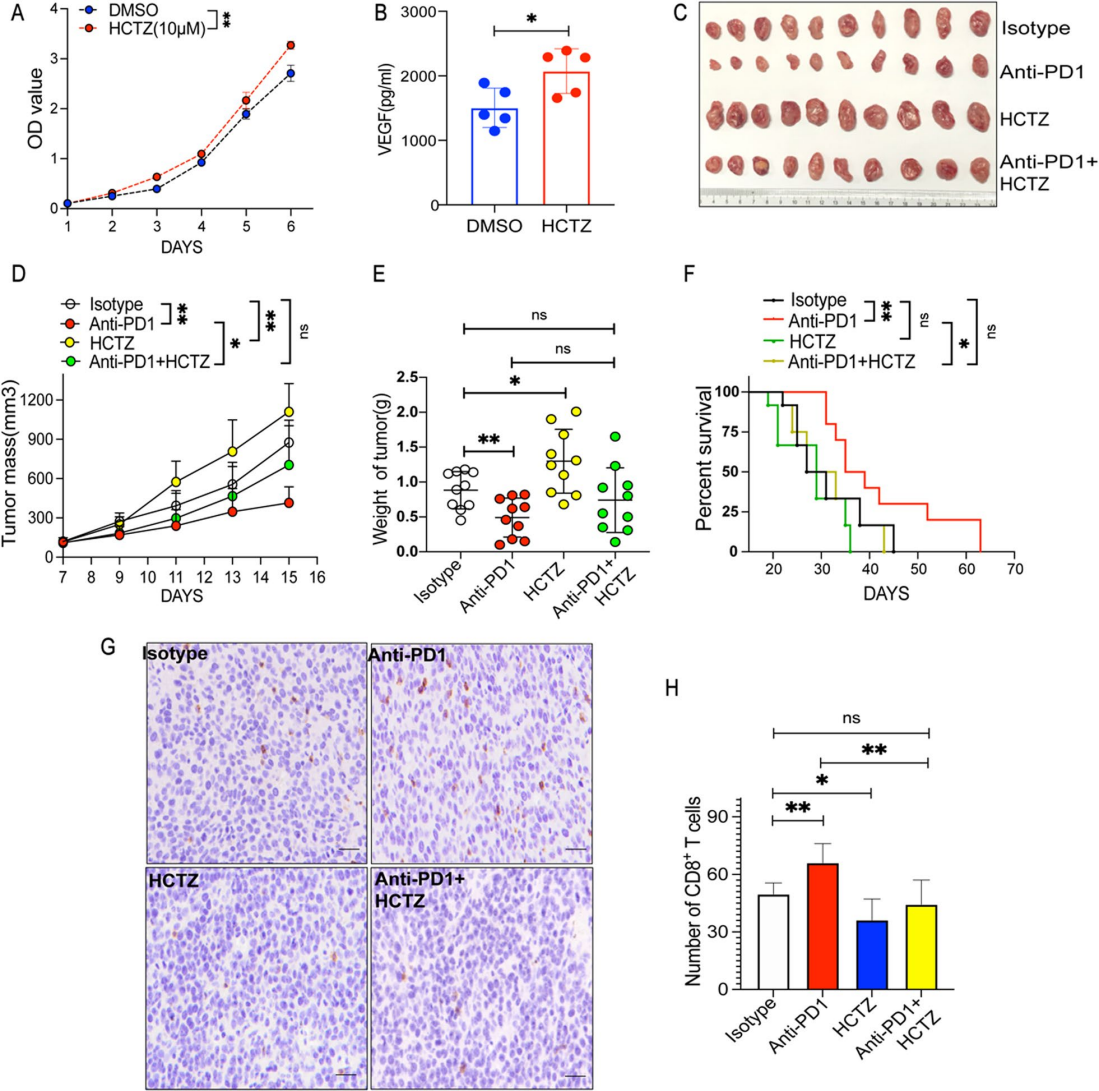
HCTZ promoted fibrosarcoma growth and weakened tumor immune responses to PD1ab. **A** CCK8 experiment was used to detect the proliferation ability of MCA-205 after HCTZ treatment. **B** Elisa kit was used to detect the VEGF production after HCTZ treatment. **C** A double therapy (HCTZ and PD1ab) or monotherapies (HCTZ or PD1ab) were used for curing MCA-205-neoplasms mice. **D**–**F** Tumor mass (**D**) and weight (**E**) from MCA-205-neoplasms mice (n = 10) were demonstrated. **F** Survival of mice (n = 10) was monitored biweekly after treatment. **G**–**H** The level of CD8^+^ T cells infiltration after treatment. Scale bars, 20 μm

As a thiazide-type diuretic, HCTZ has been used in clinical work for several decades. The drug has been used to treat hypertension globally because of its low price and safety. HCTZ works primarily by acting on the distal convoluted tubules to restrain the sodium chloride cotransporter system [33]. In vitro, HCTZ significantly promoted tumor cell growth (Fig. 6A); furthermore, it elevated the VEGF secretion of MCA-205 (Fig. 6B). In the murine subcutaneous fibrosarcoma cancer model, HCTZ promoted MCA-205 tumor growth (Fig. 6C). The combination therapy of HCTZ and PD1ab displayed a weakened inhibitory effect compared with PD1ab single therapy on MCA-205 tumor growth (Fig. 6D–E). Consequently, HCTZ alone significantly shortens the survival time of mice. Furthermore, the combination therapy of HCTZ and PD1ab displayed a weakened therapeutic impact on the survival time of mice compared with PD1ab monotherapy (Fig. 6F). Immunohistology examination of the fibrosarcoma tumors showed that HCTZ significantly decreased the number of CD8^+^ T cells. Furthermore, compared with PD1ab treatment alone, the combination of both agents resulted in a reduced level of infiltrating CD8^+^ T cells (Fig. 6G–H).

### *HCTZ enhances the proliferation and migration of human fibrosarcoma cell HT1080 *in vitro* and *in vivo

**Fig. 7:**
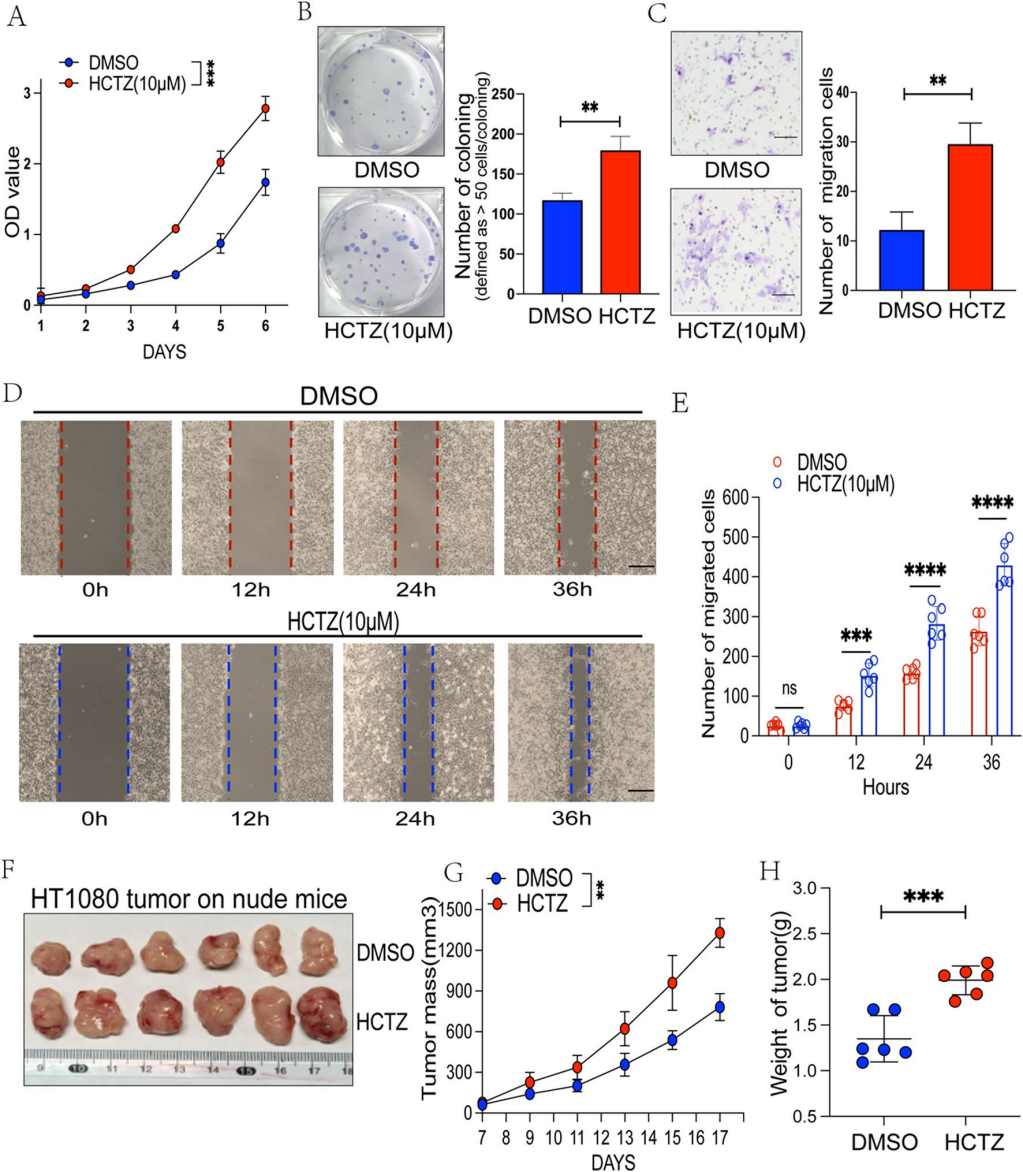
HCTZ significantly promoted human HT1080 fibrosarcoma proliferation, migration, and invasion, accelerating tumor growth in vivo*.* **A**, **B** The CCK8 detection and colony formation assays were used to analyze the effect of HCTZ on the proliferative abilities of HT1080 cells. **C** The transwell assay were used to detect the effect of HCTZ on the migrative abilities of HT1080 cells**.** Representative photographs as well as quantification are demonstrated. Scale bars, 50 μm. **D** The effect of HCTZ on the migrative abilities of HT1080 cells was examined by cell wound healing assay. Representative photographs as well as quantification are demonstrated.Scale bars, 100 μm. **E**–**G** The effect of HCTZ on tumor growth in vivo was detected by xenograft growth assay (**E**). Tumor mass (**F**) and weight (**G**) were indicated (n = 6)

Using human fibrosarcoma cell line HT1080, we further studied the effect of HCTZ in vivo and in vitro. The CCK8 and colony formation assays showed that HCTZ enhanced the viability of HT1080 (Fig. 7A–B). Moreover, HCTZ promoted the migratory abilities of HT1080, as detected by wound healing and transwell assays (Fig. 7C–D). In addition, to validate the promoting effect of tumor growth in vivo, we performed xenograft growth assays by subcutaneously injecting HT1080 cells into nude mice (Fig. 7E). The xenograft tumors from the HCTZ-treated group were more vigorous than those from the control group (Fig. 7F–G). The results indicated that HCTZ promotes the tumorigenesis of human fibrosarcoma cell HT1080 in vitro and in vivo.

### HCTZ promoted proliferation and tumorigenesis in vivo and in vitro through SLC12A3

**Fig. 8:**
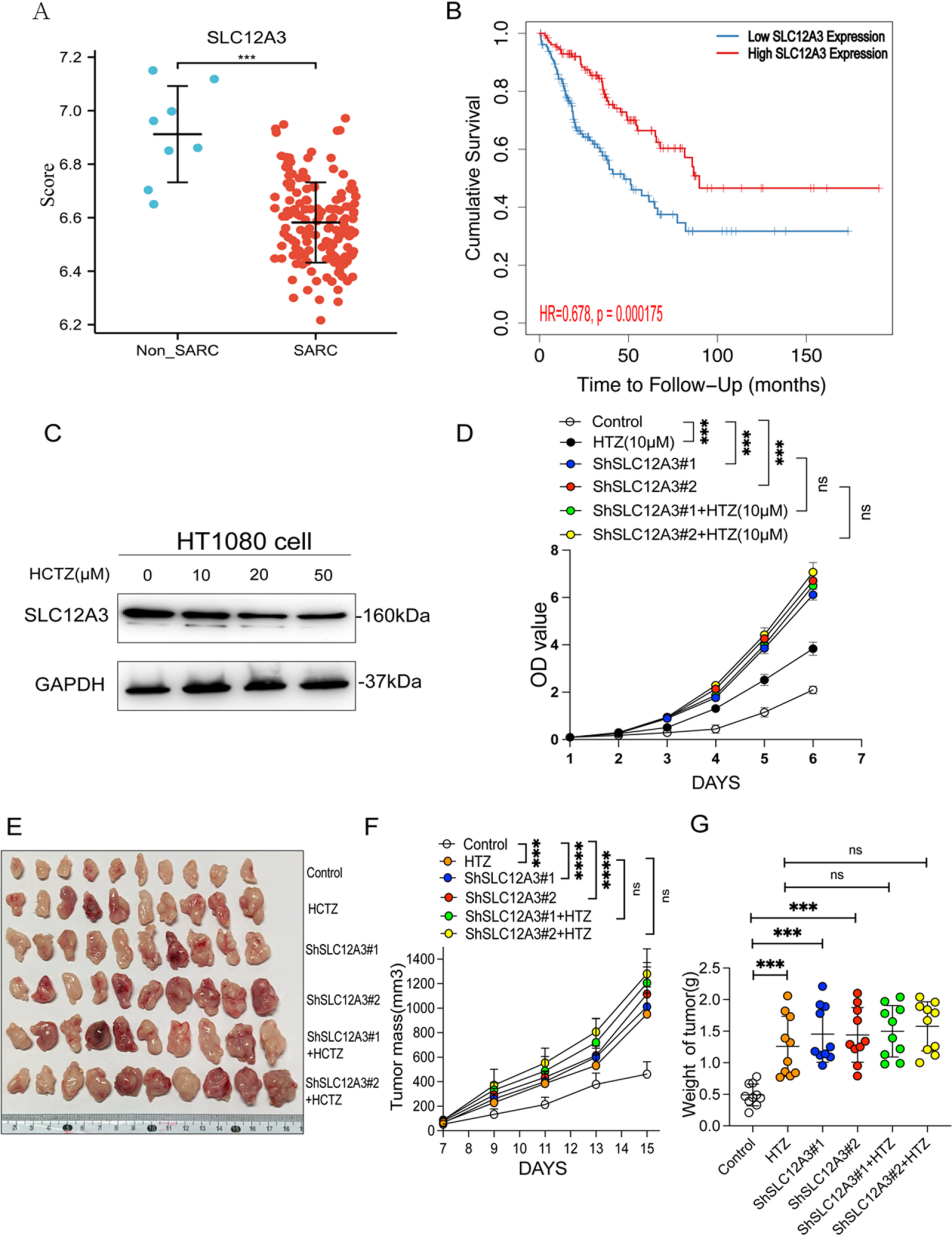
Hydrochlorothiazide exerts its promoting effect through SLC12A3. (**A–B**) Data from the GEO were utilized to analyze the association between overall survival and SLC12A3 expression in sarcoma patients. (**C**) WB analysis of the SLC12A3 protein level in HT1080 after applying different concentrations of HCTZ. (**D**) The CCK8 assay detected the proliferation ability of HT1080 (shRNA#1 and shRNA#2) when adding HCTZ. (**E–G**) The xenograft growth assay indicated the tumor mass (**F**) and weight (**G**) of the HT1080 tumor (shRNA#1 and shRNA#2) when treated with HCTZ

SLC12A3 was recognized as a target of HCTZ, which encodes a sodium-chloride cotransporter that regulates Na^+^ and Cl^–^ balance in the distal renal convoluted tubule [34]. It is difficult to determine whether SLC12A3 plays a role in the promotion of tumor progression by HCTZ. Analysis of Gene Expression Omnibus(GEO) data (GSE21122) showed that SLC12A3 is lower in sarcoma tissues than in normal tissues (Fig. 8A). Additionally, by using the GEPIA2 web tool, the poor prognosis of sarcoma patients showed a close association with the high expression of SLC12A3 (Fig. 8B). The protein concentration of SLC12A3 in HT1080 cells treated with varying concentrations of HCTZ was determined by Western blotting. HCTZ introduction reduced the protein level of SLC12A3 in a dose-dependent manner (Fig. 8C). To validate the mechanism of HCTZ, a lentiviral vector carrying shRNA-targeted endogenous SLC12A3 was successfully employed. When SLC12A3 was knocked down in HT1080 cells in vitro, the shSLC12A3 and HCTZ groups demonstrated a greater proliferative capacity than WT cells. However, compared with shSLC12A3 group, the shSLC12A3 plus HCTZ showed no significant promoting effect (Fig. 8D). Consistent results were observed in xenograft growth assays, and HCTZ treatment alone and knockdown of SLC12A3 promoted human fibrosarcoma growth. When SLC12A3 was knocked down, HCTZ treatment did not improve tumor promotion (Fig. 8E–G). These results preliminarily demonstrated that HCTZ might promote tumor progression via SLC12A3.

## Discussion

Many tumor patients are co-diagnosed with hypertension. Therefore, it is crucial to investigate the relationship between anti-hypertensive medications and tumor immunotherapy. This research has at least three advantage: (1) It can offer guidance to hypertension patients undergoing tumor immunotherapy regarding selecting more appropriate anti-hypertensive drugs. (2) Combining anti-hypertensive drugs and tumor immunotherapy may provide new therapeutic strategies for cancer patients with or without hypertension. (3) It could aid the clinician in avoiding the use of anti-hypertensive drugs that promote tumor growth. In this study, we investigated the effects of six widely used anti-hypertensive drugs, including verapamil, losartan, spironolactone, HCTZ, captopril, and furosemide, on tumor growth with the PD1ab immune checkpoint inhibitor. We discovered that various anti-hypertensive medications have wide and varied effects on tumor growth. Verapamil suppressed tumor growth and displayed an enhancing impact on the PD1ab therapy. Captopril did not affect tumor growth but brought an unexpected benefit to PD1ab treatment. In contrast, spironolactone and furosemide showed no effect on tumor growth but had an offset effect on the PD1ab therapy. Alarmingly, losartan and HCTZ, especially HCTZ, promoted tumor growth and weakened the effect of PD1ab treatment. Furthermore, we preliminarily found that HCTZ may promote tumor progression through SLC12A3.

Verapamil is considered one of the most effective and safe drugs in the dihydropyridine L-type calcium channel blocker class by the World Health Organization (WHO). Most research on verapamil treatment of tumors has centered on drug resistance, which has been reported to inhibit the growth of various tumors, including lung as well as breast cancer [30, 35–37]. A retrospective cohort study and meta-analysis reported an increased risk of lung, skin, and breast cancer in verapamil users [38–41]. However, it remains controversial as some cohort studies have demonstrated that using verapamil does not increase the risk of cancer [42–45] or reduce it [46]. However, it was recently reported that verapamil inhibited the in vitro activation and function of T lymphocytes [47]. The precise functions that verapamil will perform in immunotherapy remain unknown. This study, consistent with the outcomes observed in numerous other types of cancer, demonstrated that verapamil significantly inhibited the growth of fibrosarcoma and showed an enhanced effect on PD1ab, which may indicate that it possesses a favorable anti-tumor growth ability. Furthermore, it exhibited a significantly enhanced anti-tumor impact of PD1ab therapy, which aligns with the findings of a recent study reporting that the analogous anti-hypertensive nifedipine potentiated PD1ab therapy on CRC [48]. Therefore, we strongly recommend verapamil as the treatment of choice to control blood pressure when fibrosarcoma patients are co-diagnosed with hypertension. Furthermore, the combination of verapamil and PD1ab might be utilized as a novel strategy for treating other malignancies, regardless of whether the patient has hypertension. More detailed researches and clinical trials should be conducted in the future.

Captopril is an ACEI agent widely used in hypertensive patients. The effect of captopril on tumor progression remains quite unclear. In a related report, captopril was claimed to improve the therapeutic effect on multi-type cancer, including but not limited to depleting the excess extracellular matrix in pancreatic tumors to help the entry of chemotherapeutic agent gemcitabine [49], softening liver metastases, and improving the anti-angiogenic properties of bevacizumab in hepatic carcinoma [50–52]. Conversely, captopril was demonstrated to accelerate the growth of immunogenic RenCA tumors, MethA sarcoma [14], and S-180 sarcoma [13] in the murine model. Captopril was also reported to increase PD-1 expression on infiltrating CD8^+^ T cells in a murine model [53, 54]. However, the effect of the combination of captopril and PD1ab on tumor growth remains unclear. In different clinical studies, the use of captopril was reported to increase [55–60], decrease [61, 62], or have no effect [63, 64] on the risk of multi-type cancer. In this study, the combination therapy of captopril and PD1ab showed an unexpectedly enhanced inhibitory effect on MCA-205 growth compared with PD1ab single therapy, consistent with the outcome reported in CRC [54]. Accounting for tumor heterogeneity, a detailed study of captopril in fibrosarcoma should be conducted in the future. Under PD1ab therapy, we suggest that captopril should be prescribed more frequently, particularly for fibrosarcoma patients.

Losartan is an angiotensin II receptor antagonist extensively used in hypertensive patients. In vitro and in vivo experiments displayed that losartan prevents colorectal cancer [65, 66], breast cancer [67], osteosarcoma [68] and ovarian cancer [69] progression in mice. In addition, losartan can enhance PD1ab therapy efficacy in breast cancer [70]. Some researchers [71] claimed that losartan could increase cancer risk by eliminating AT1R "competition" and increasing AT2R-mediated angiogenesis in vivo. While some scholars hold different opinions that losartan decreases the chances of cancer [72]. A retrospective cohort study illustrated that losartan could increase [19], decrease [67, 73] or produce a neutral effect on the risk of tumor [20]. In this study, losartan monotherapy promoted the growth of MCA-205 tumors in vivo. Compared with PD1ab single therapy, the combination therapy of losartan and PD1ab did not improve the therapeutic effect on MCA-205 tumor inhibition. It is inconsistent with the result that losartan could enhance PD1ab inhibition in the breast cancer model [70], which may remind us that the dose and frequency of losartan and cancer type should not be should not be neglected.

Furosemide is one of the loop diuretics broadly used in clinical work. There is no report about the furosemide role in PD1ab combination on tumor inhibition. Data from the UK Cancer Registry Study showed that furosemide was unrelated to improved prognosis in gastric or esophageal cancer patients [74]. It is more or less consistent with our results that furosemide showed no effect on fibrosarcoma growth and reduced tumor immune responses to PD1ab.

Spironolactone is one of the mineralocorticoid receptor antagonists used in managing and treating hypertension. In cell experiments, spironolactone was considered to boost anticancer effects by inhibiting DNA damage repair in osteosarcoma and cervical cancer [17]. The role of spironolactone in PD1ab combination on tumor inhibition is understudied. From a case–control study in Sweden, patients who used spironolactone had a reduced risk of prostate cancer, and the risk of prostate cancer decreased as the dose increased [75]. Our study found that compared with PD1ab single therapy, the combination therapy of spironolactone and PD1ab impaired the tumor inhibition on fibrosarcoma growth, neglecting its neutral effect on fibrosarcoma growth. Spironolactone was once reported to reduce vital protein CD62L and CXCR4 expression in T cells[76], which may give a hint that spironolactone may directly affect the status of T cell and thus impaired the therapeutic effect of PD1ab. However, the hypothesis should be tested by adequate experiments in the future.

HCTZ is the thiazide-type diuretic largely employed as the first-line treatment of hypertension. There are few reports about the HCTZ role in tumor progression in vivo and in vitro. According to some evidence, the use of HCTZ is associated with an increased risk of various skin cancers, including basal cell carcinoma, cutaneous squamous cell carcinoma, malignant melanoma, and lip cancer among the European and American population [77–79]. Moreover, the use of HCTZ seems to be safe for skin cancer risk among the Asian population [80], and a lower absolute risk was found in the Korean population [81]. In this study, we discovered that HCTZ alone stimulated fibrosarcoma growth and diminished the therapeutic effect of PD1ab. HCTZ was proved to be preventing vascular T-cell infiltration in tissue in condition of high blood pressure[82], which may also reduce the infiltration of CD8^+^T cells in tumor microenvironment by alter the vascular permeability or secretion of chemokines and so on. The detailed study should be conducted in the future. Consequently, HCTZ alone significantly shortened the survival time of mice. Furthermore, in vivo* and *in vitro experiments, HCTZ promoted proliferation, migration and tumorigenesis of human fibrosarcoma cells.

SLC12A3 is considered to encode a renal thiazide-sensitive sodium-chloride cotransporter, recognized as the target for thiazide diuretics to control high blood pressure. Employing weighted gene co-expression network analysis (WGCNA), lower SLC12A3 expression in tumors was found to correlate with shorter survival in patients with kidney renal clear cell carcinoma (KIRC) [83], consistent with our findings. SLC12A3 may contribute to the radio-resistance of lung cancer [84]. The role of SLC12A3 in tumor progression still largely needs to be explored. In this research, low expression of SLC12A3 was associated with a worse prognosis in sarcoma. HCTZ introduction reduced the expression of SLC12A3 in a dose-dependent way. By successful knockdown of SLC12A3, HCTZ did not exhibit the promoting effect on the tumor in vivo and in vitro. These results demonstrated that HCTZ could promote tumor progression via SLC12A3. Further mechanism studies should be put into practice in the future.

In conclusion, we proposed that in treating fibrosarcoma with PD1ab, hypertensive patients should choose verapamil and captopril to stabilize blood pressure, thereby enhancing the therapeutic effect of PD1ab on fibrosarcoma. Furosemide and spironolactone are not advised because they may diminish the anti-fibrosarcoma effect of PD1ab. Most importantly, HCTZ must be utilized with extreme caution (Table 1). In the future, futher mechanism studies and clinical trials should be conducted. We hope that numerous tumor patients, particularly those with fibrosarcoma, will benefit from these studies.

**Table 1:**
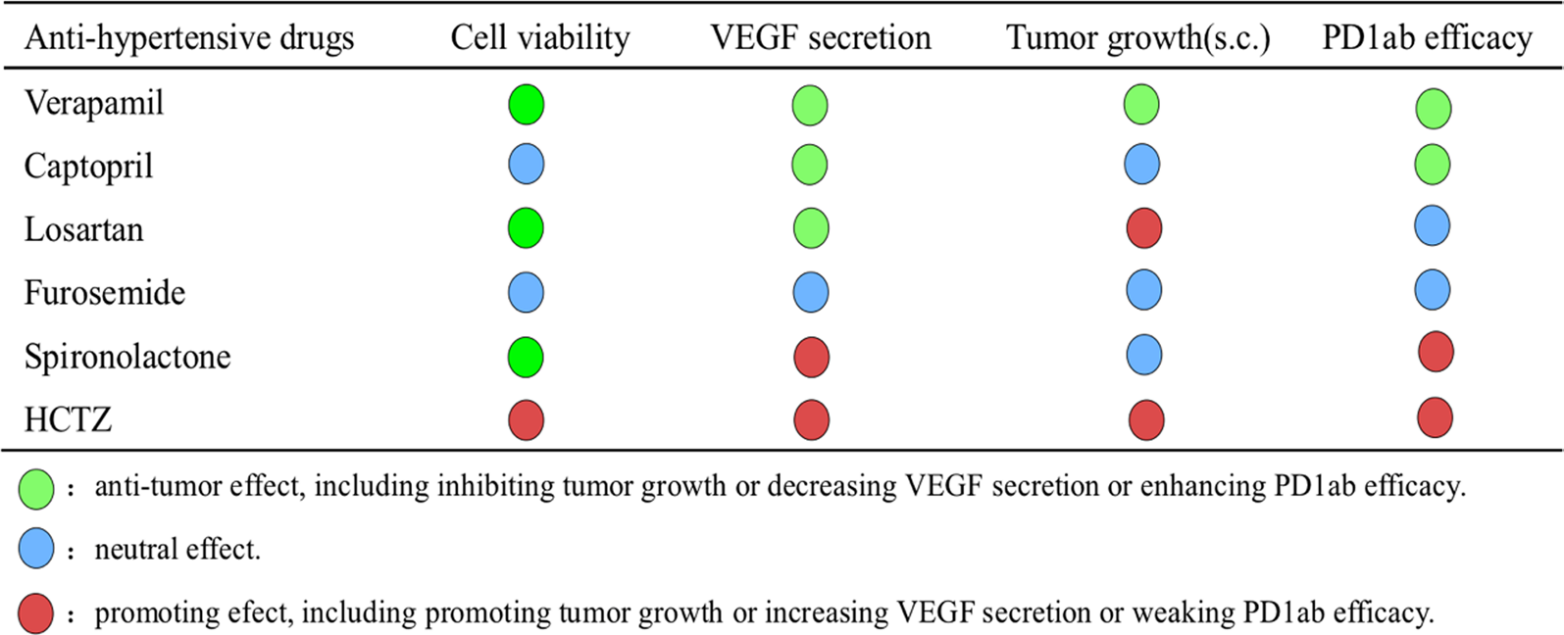
Six anti-hypertensives act on fibrosarcoma progression and PD1ab blockade therapy

## Data Availability

All data are available in the main text or the supplementary materials.
